# Adrenal Ganglioneuroma Masquerading as a Suspicious Adrenal Incidentaloma: A Case Report and Review of the Literature

**DOI:** 10.7759/cureus.82455

**Published:** 2025-04-17

**Authors:** Zein B Sheikh, Nariman A Nawar, Abdulrahman S Alamri

**Affiliations:** 1 Internal Medicine, Prince Mohammed Bin Abdulaziz National Guard Hospital, Al Madinah, SAU; 2 Pathology, Prince Mohammed Bin Abdulaziz National Guard Hospital, Al Madinah, SAU; 3 Endocrinology and Metabolism, Prince Mohammed Bin Abdulaziz National Guard Hospital, Al Madinah, SAU

**Keywords:** adrenal, case report, ganglioneuroma, incidentaloma, tumor

## Abstract

Adrenal ganglioneuromas are rare tumors. Although benign in nature, radiographic features are non-diagnostic and can be suspicious for malignant tumors, necessitating surgical adrenalectomy for histopathologic diagnosis. Most are discovered incidentally, and are hormonally inactive. Workup to exclude functional tumors is warranted. We describe a case of a 58-year-old female patient with an adrenal ganglioneuroma masquerading as a suspicious adrenal incidentaloma on imaging.

## Introduction

Ganglioneuromas (GNs) are rare benign tumors originating from the sympathetic chain. They are composed of mature ganglion cells, Schwann cells, and nerve fibers [1]. Commonly, GNs are found in the posterior mediastinum and retroperitoneum. Rarely, they can originate from the adrenal glands [2]. The majority of these tumors are hormonally inactive and discovered incidentally on abdominal imaging done for unrelated reasons [3]. A small number of reported cases are secretory, mainly of catecholamines, which can be confused with pheochromocytoma [4]. The incidence of adrenal GNs is unknown; however, with the advances of imaging techniques and their increasing accessibility, more cases are being recognized [5-7]. Although these tumors are benign in nature, they usually undergo surgical resection for a suspicion of malignant tumors, as there is a lack of definitive radiographic diagnostic features [6]. Here, we describe a case of a 58-year-old female patient with a suspicious-looking adrenal incidentaloma, which was recognized as a GN upon postoperative histopathologic evaluation. This report presents the clinical and radiological data of this rare tumor, and reviews the literature to facilitate the diagnosis of adrenal GNs.

## Case presentation

**Figure 1:**
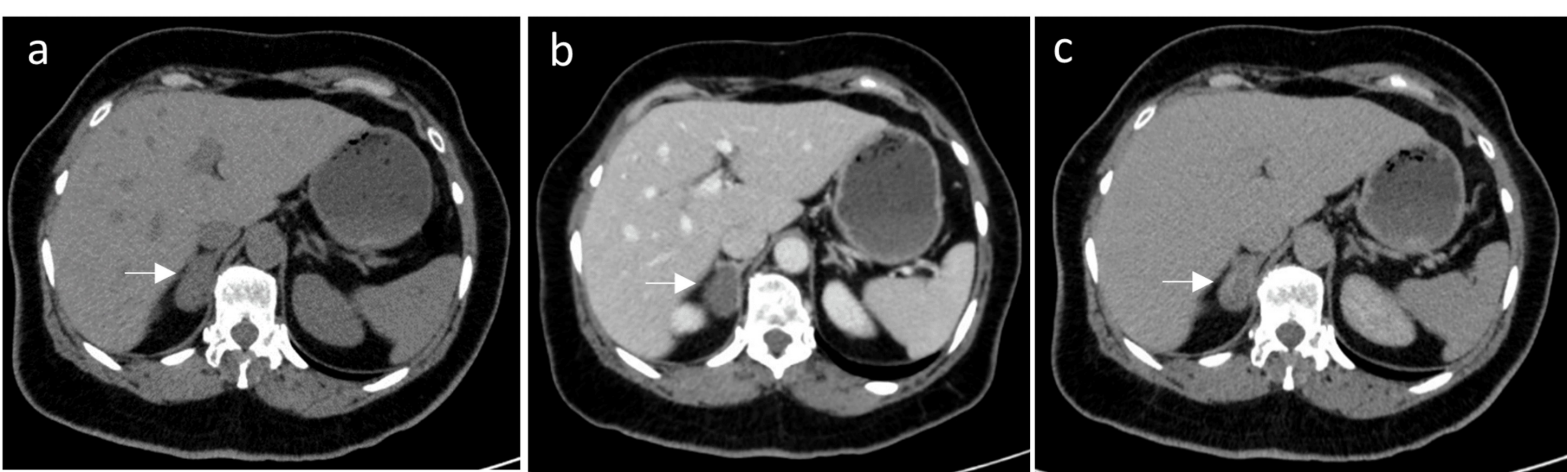
Computed tomography scan of the abdomen with adrenal protocol. Computed tomography scan of the abdomen with adrenal protocol showing a right adrenal mass (Arrow) measuring 3.2 x 1.8 cm in the a) pre-contrast phase, b) portovenous phase, and c) delayed phase.

A 58-year-old woman with a history of non-obstructive renal stones diagnosed via ultrasonography a few years earlier reported experiencing recurrent renal colic over the past few months and was referred to urology for further evaluation. She underwent an unenhanced computed tomography (CT) scan of the kidneys, ureters, and bladder, which showed a left lower pole nonobstructive renal stone measuring 0.8 cm. In addition, an incidental finding of a lesion arising from the medial limb of the right adrenal gland was identified. The mass measured 2.6 cm, was homogenous, isodense to muscle, with a mean density of 22 Hounsfield units (HUs), no calcification, and no cystic or hemorrhagic component was seen, necessitating further workup with CT scan of the abdomen with adrenal protocol. A new CT scan showed a right adrenal mass measuring 3.2 x 1.8 cm, with pre-contrast HUs of 32, 37 HUs in the venous phase, and 54 HUs in the delayed phase (Figure 1a-1c). It had an absolute contrast washout of -340%, and was considered to be an indeterminate lesion based on the American College of Radiology criteria for management of incidental adrenal masses. There was no evidence of a metastatic disease on CT scan.

She was referred to endocrinology for evaluation of this adrenal incidentaloma. Medical history included type II diabetes mellitus on metformin, primary hypertension well controlled on candesartan alone, and dyslipidemia on atorvastatin. Patient denied any history of palpitations, headache, sweating, or weight gain. Family history was noncontributory. On physical examination, her blood pressure was 132/60 mmHg, and her heart rate of 64 beats per minute. There were no clinical features of Cushing’s syndrome. Workup to exclude functioning adrenal mass included: 1 mg overnight dexamethasone suppression test, aldosterone/renin ratio, and 24 h urine metanephrines and normetanephrines. Laboratory results came back negative for subclinical Cushing’s syndrome, hyperaldosteronism, and pheochromocytoma (Table 1).

**Table 1:** Laboratory results for hormonal workup.

Test	Result	Reference range
Renin	15.9 ulU/ml	4.2-59.7 ulU/ml
Aldosterone	<3.7 ng/dl	<31.0 ng/dl
24 h urine metanephrine	0.32 umol/24 h	<1.8 umol/24 h
24 h urine normetanephrine	0.54 umol/24 h	<3.0 umol/24 h
1 mg overnight dexamethasone suppression test	31 nmol/L	<55 nmol/L

Given the indeterminate imaging features, malignancy couldn't be ruled out. After discussion with the patient, the decision was taken to remove the tumor. The patient was referred to surgery and underwent an uneventful laparoscopic right adrenalectomy. 

Pathology

Gross Description

The resected specimen consisted of a 3.4 x 3.0 x 1.5 cm oval mass and an unremarkable adrenal gland. The mass abuts the adrenal gland cortex, with no gross infiltration. It is tan-white in color and firm with homogeneous consistency. No necrotic or cystic areas were identified.

*Microscopic Description * 

**Figure 2:**
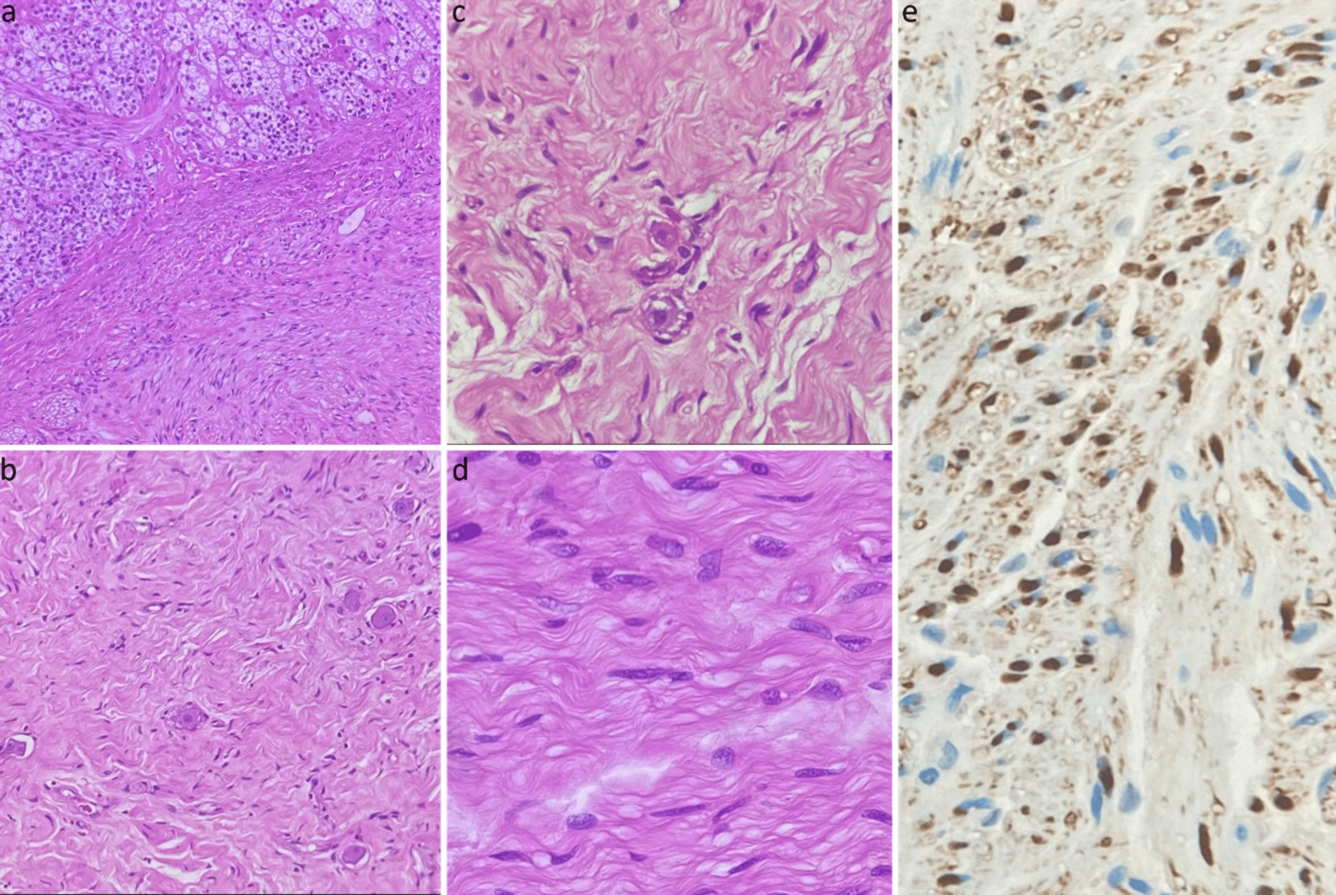
Microscopy of adrenal ganglioneuroma. (a) Interface between adrenal gland cortex and tumor (20x, H&E stain), (b) dispersed ganglion cells in Schwannian stroma (20x, H&E stain), (c) high power displaying ganglion cells in various stages of maturation (40x, H&E stain), (d) Schwannian rich stroma (40x, H&E stain), and (e) S100 immunostain highlighting Schwannian rich stroma (40x).

Sections demonstrated a neoplasm consisting of spindle cells and ganglion cells. The spindle cell component is the predominant component. They are wavy, with pale pink cytoplasm and bland nuclei. Those cells were positive for S100 immunostain, which aided in characterizing them as Schwann cells. The ganglion cells are scattered throughout the tumor, of different sizes and shapes, and display various stages of maturation. No small blue cell component or neuropil is identified. Based on the described features, the neoplasm was diagnosed as ganglioneuroma, maturing subtype (Figures 2a-2e).

Repeated postoperative scans show no evidence of residual mass. The patient is on annual follow-up without evidence of recurrence.

## Discussion

In this report, we describe a case of a 58-year-old female patient with a suspicious adrenal incidentaloma, diagnosed as a GN upon post-resection histopathologic evaluation. GNs are a rare finding in adrenalectomy specimens. These tumors are benign in nature, originating from the neural crest tissue in the sympathetic nervous system [8]. As in the present report, these tumors are mostly silent and are discovered incidentally during abdominal imaging done for another reason [9]. A minority of reported cases are secretory of catecholamines and androgens, thus excluding hormonal activity is essential [10]. Others present with compressive symptoms of abdominal discomfort or non-specific abdominal pain due to their growing size [11,12]. The appearance of these tumors on imaging is diverse and lacks pathognomonic features, which is a cause of diagnostic challenge preoperatively [13]. The misdiagnosis rate based on imaging has been reported to be up to 64.7% [14]. The non-specific computed tomography (CT) scan findings include a homogenous, well-circumscribed hypodense mass in the pre-contrast phase, and homogenous to heterogenous hyperdense enhancement in the late phase. Additionally, macro/microcalcifications are seen with variable incidence ranging from 11.1% up to 82.4%. Some of these features are similar to malignant adrenal tumors, thus increasing their chance of being labeled as a suspicious lesion prior to excision [15].

Similar cases have been published in the literature. Lee et al. reported the largest case series of adrenal GNs in a single institution experience. In their retrospective review of 1784 patients who underwent adrenalectomy, 35 adrenal GNs were identified (1.9%). One of the most consistent CT scan findings was post-contrast Hounsfield units (HUs) higher than the pre-contrast values, similar to our case. Nonetheless, this is not specific to adrenal GNs, but a consistent finding of neurogenic tumors [7]. Sasaki et al. described an adrenal GN case in a 41-year-old man that was highly suspicious of adrenocortical carcinoma upon assessment with CT and positron emission tomography (PET) scans [2]. Similarly, in a case series of four patients by Johnson et al., all cases underwent surgical adrenalectomy for suspicious tumors on imaging [3]. Kayashta et al. reported a case of an adrenal GN with a differential diagnosis based on CT scan of adrenal lymphangioma, GN, and a nerve sheath tumor [1]. A case report by Burns et al. described an adrenal GN case with high levels of 24-hour urine dopamine, misdiagnosed as a dopamine-secreting pheochromocytoma prior to surgical excision [4]. Some cases have been misdiagnosed on pre-operative biopsies as well. A case report by Sadek et al. described an adrenal GN in a 27-year-old man who underwent a CT scan-guided biopsy of a large left adrenal mass revealed an inconclusive diagnosis of a spindle cell tumor vs a gastrointestinal stromal tumor. A diagnosis of adrenal GN was confirmed post-laparoscopic resection [14]. In contrast to our case, most cases of adrenal GNs are of large size, ranging from 5 to 13 cm [3,6], and in one report up to 22 cm [2]. Although the adrenal mass size in the present study was not large, the imaging finding of an absolute contrast washout <60% in the delayed CT scan phase was considered indeterminate and required surgical resection to rule out carcinoma.

## Conclusions

Adrenal GNs, although benign tumors, are usually resected during evaluation for a suspicious adrenal mass. Predominantly, these tumors are hormonally silent, while a minority secrete catecholamines or androgens. The non-specific imaging appearance provides a challenge in preoperative diagnosis prior to histopathologic evaluation. We recommend considering GNs as a differential diagnosis of adrenal incidentalomas with calcifications on imaging. We encourage the publication of such cases to determine if any homogeneity exists in radiographic appearance, thus avoiding unnecessary invasive surgical procedures.
